# Anti-Cancer Potential of Edible/Medicinal Mushrooms in Breast Cancer

**DOI:** 10.3390/ijms241210120

**Published:** 2023-06-14

**Authors:** Marzia Bruna Gariboldi, Emanuela Marras, Nicole Ferrario, Veronica Vivona, Pamela Prini, Francesca Vignati, Gianpaolo Perletti

**Affiliations:** Department of Biotechnology and Life Sciences (DBSV), University of Insubria, 21100 Varese, Italy; emanuela.marras@uninsubria.it (E.M.); nferrario1@studenti.uninsubria.it (N.F.); vvivona@studenti.uninsubria.it (V.V.); pamela.prini@uninsubria.it (P.P.); vignati.francesca@gmail.com (F.V.); gianpaolo.perletti@uninsubria.it (G.P.)

**Keywords:** breast cancer, edible/medicinal mushrooms, in vitro studies, in vivo studies, clinical studies

## Abstract

Edible/medicinal mushrooms have been traditionally used in Asian countries either in the cuisine or as dietary supplements and nutraceuticals. In recent decades, they have aroused increasing attention in Europe as well, due to their health and nutritional benefits. In particular, among the different pharmacological activities reported (antibacterial, anti-inflammatory, antioxidative, antiviral, immunomodulating, antidiabetic, etc.), edible/medicinal mushrooms have been shown to exert in vitro and in vivo anticancer effects on several kinds of tumors, including breast cancer. In this article, we reviewed mushrooms showing antineoplastic activity again breast cancer cells, especially focusing on the possible bioactive compounds involved and their mechanisms of action. In particular, the following mushrooms have been considered: *Agaricus bisporus*, *Antrodia cinnamomea*, *Cordyceps sinensis*, *Cordyceps militaris*, *Coriolus versicolor*, *Ganoderma lucidum*, *Grifola frondosa*, *Lentinula edodes*, and *Pleurotus ostreatus*. We also report insights into the relationship between dietary consumption of edible mushrooms and breast cancer risk, and the results of clinical studies and meta-analyses focusing on the effects of fungal extracts on breast cancer patients.

## 1. Introduction

Breast cancer (BC) is among the major causes of cancer-related deaths worldwide, with 2.3 million new cases per year, according to the GLOBOCAN 2020 data [1]. Projections indicate that by 2030, the number of new cases diagnosed worldwide will reach 2.7 million, while the number of deaths will reach 0.9 million [2] (Global Cancer Observatory: Cancer Tomorrow, accessed on 29 April 2023. Available online: https://gco.iarc.fr/tomorrow). In addition, the incidence of breast cancer is expected to further increase, in particular in low- and medium-income countries, due to the effects of a westernized lifestyle, characterized by delayed pregnancies, reduced breastfeeding, low age at menarche, lack of physical activity, and poor diet [3].

Breast cancer is categorized into three major subtypes based on the presence or absence of molecular markers for estrogen, progesterone, and human epidermal growth factor 2 (ERBB2, also known as HER2) receptors: hormone receptor positive/HER2 negative (70% of patients), HER2 positive (15–20%), and triple-negative (TNBC, tumors lacking all three standard molecular markers; 15%) [4,5].

A number of BC risk factors have been established, including family history. This association is driven by epigenetic changes as well as by environmental factors acting as potential triggers [6]. In addition, several genetic mutations have been highly associated with an increased risk of BC. Particularly, *BRCA1* and *BRCA2* are two major genes primarily linked to the increased risk of breast carcinogenesis [7]. Other highly penetrant BC genes include *TP53*, *CDH1*, *PTEN*, *CHECK2*, and *STK11* [8,9,10,11]. According to recent research, mutations within the *XRCC2* gene could also be potentially associated with an increased risk of breast cancer [12].

According to the World Health Organization (WHO), alcohol, highly processed meat consumption, and excessive intake of saturated fats might enhance the risk of both gastrointestinal tract tumors and BC [13,14]. Ultra-processed food is rich in sodium, fat, and sugar, which subsequently predispose to obesity, recognized as another factor of BC risk [15]. It was observed that a 10% increase in ultra-processed food in the diet is associated with an 11% greater risk of BC. Conversely, a diet rich in vegetables, fruits, legumes, whole grains, and lean protein is associated with a lowered risk of BC [16]. Generally, a diet that includes food containing high amounts of ω-3 PUFA, vitamin D, fiber, folate, and phytoestrogen might be beneficial to prevent BC [17]. In addition, a lower intake of n-6 PUFA and saturated fat is recommended.

Conventional approaches, including surgery, chemotherapy, immunotherapy, radiotherapy, targeted therapy, and endocrine therapy, are routinely used to treat BC. Although the treatment of patients with BC has evolved dramatically over the past decades, the median overall response is still poor. In particular, triple-negative BC remains rather untreatable and, as with other tumors, BC cells may undergo acquired resistance, which reduces the tumor’s response to conventional treatments [18]. In addition, these treatments may lead to severe adverse effects such as myelosuppression, gastrointestinal disorders, hemorrhagic cystitis, alopecia and cardio- and neuro-toxicity, which limit the patient’s quality of life [19].

In recent years, the impact of the adverse effects of conventional therapy, as well as the poor results obtained with conventional medicinal approaches, highlighted the need for alternative therapeutic strategies. Among the complementary and integrative approaches evaluated, the combination of conventional medicine and complementary activities, including the use of a wide range of products, such as herbs, vitamins, nutraceuticals, and probiotics, has shown promising results [20,21,22]. In this context, edible/medicinal mushrooms have emerged not only as sources of new nutraceuticals but also as a possible complementary and alternative medicine, showing interesting results as an adjuvant to conventional chemo- or radiation-therapy, enhancing their potency or reducing their side effects, thus improving the patient’s quality of life [23,24].

The antineoplastic effects of mushrooms have been mainly related to their ability to modulate the immune system, thanks to their content in glucans, sesquiterpenes, glycoproteins, or peptide/protein-bound polysaccharides [23,25]. Furthermore, minerals, amino acids, other organic compounds, and several vitamins (e.g., thiamin, riboflavin, ascorbic acid, and vitamin D) are contained in mushrooms and contribute to their overall health benefits [25]. Some of these natural mushroom compounds have demonstrated specific activity against signaling pathways that are aberrantly activated in cancer cells and have been shown to negatively modulate specific molecular targets involved in cell proliferation, survival, and angiogenesis [26,27]. The potential therapeutic effects of mushrooms have been investigated both at the preclinical and clinical levels. Several quality reviews published in recent years have summarized the effects of mushrooms and mushroom extracts in human disease and, more specifically, in cancer [28,29,30].

**Figure 1:**
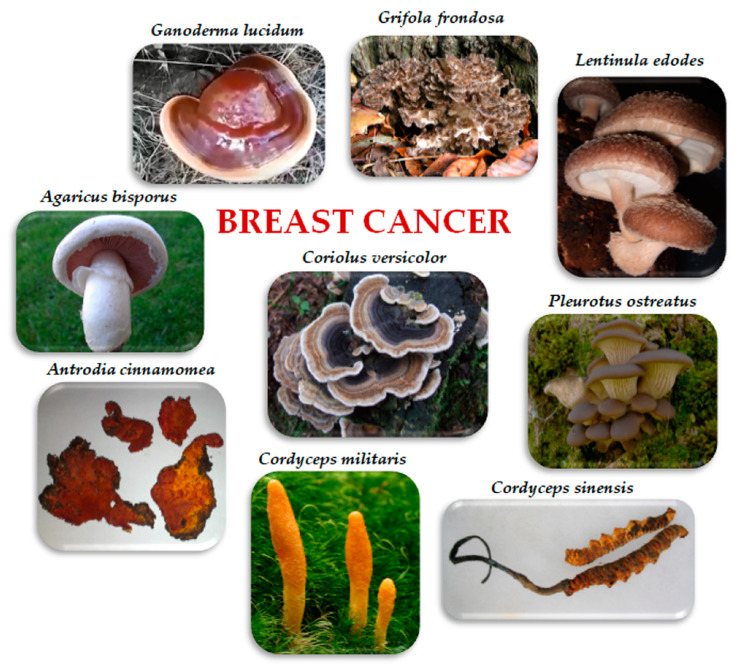
Edible/medicinal mushrooms active in breast cancer considered in this review (credits: *Agaricus bisporus*, by Jerzy Opioła, Own work, CC BY-SA 3.0, https://commons.wikimedia.org/w/index.php?curid=24843662; *Antrodia cinnamomea*, by Thomaswz19, Own work, CC BY-SA 3.0, https://commons.wikimedia.org/w/index.php?curid=30570855; *Cordyceps militaris*, By Andreas Kunze, Own work, CC BY-SA 3.0, https://commons.wikimedia.org/w/index.php?curid=16244069; *Cordyceps sinensis*, by L. Shyamal, Own work, CC BY-SA 3.0, https://commons.wikimedia.org/w/index.php?curid=4116391; *Coriolus versicolor*, by Jerzy Opioła, Own work, CC BY-SA 3.0, https://commons.wikimedia.org/w/index.php?curid=25175312; *Ganoderma lucidum*, by Shane Hanofee, Attribution, https://en.wikipedia.org/w/index.php?curid=64332594; *Grifola frondosa*, by Pethan, CC BY-SA 3.0, https://commons.wikimedia.org/w/index.php?curid=1792907; *Lentinula edodes*, by frankenstoen from Portland, Oregon, Shiitake Mushrooms, CC BY 2.0, https://commons.wikimedia.org/w/index.php?curid=7304024; *Pleurotus ostreatus*, by Archenzo, Own work, CC BY-SA 3.0, https://commons.wikimedia.org/w/index.php?curid=3005251, accessed on 29 April 2023).

This review aimed to summarize the described in vitro and in vivo effects of edible/medicinal mushrooms, which have been investigated for their direct or indirect activity on breast cancer. In particular, we considered *Agaricus bisporus* (J.E. Lange) Imbach, *Antrodia cinnamomea* T.T. Chang and W.N. Chou, *Cordyceps sinensis* (Berk.) Sacc. and *Cordyceps militaris* (L.) Fr., *Coriolus versicolor* (L.) Quél., *Ganoderma lucidum* (Curtis) P. Karst., *Grifola frondosa* (Dicks.) Gray, *Lentinula edodes* (Berk.) Plegrel, and *Pleurotus ostreatus* (Jacq.) P. Kumm. (Figure 1). We report insights about the mechanisms through which they exert antitumoral effects on BC. We also review studies and meta-analyses about the effects of edible/medicinal mushroom extracts on breast cancer patients, as well as the effect of dietary consumption of mushrooms on breast cancer risk.

## 2. Bioactive Compounds in Medicinal Mushrooms and Their Mechanisms of Action

Mushrooms’ bioactivities have been related to several biologically active compounds, including polysaccharides, which are structural components of the fungal cell wall. Polysaccharides have been shown to exert antitumor, immunomodulatory, antioxidant, anti-inflammatory, antimicrobial, and antidiabetic activities [31,32,33]. However, specific chemical features, such as the weighted degree of branching, backbone linkage, side-chain units, and the type of constituent monosaccharides, can influence the type and modulation of these biological activities. α- and β-glucans are the most abundant polysaccharides, whereas other glycans, such as heteroglycans, peptidoglycans, and polysaccharide–protein complexes, are known to exert important biological activities [31,34].

Mushrooms are rich in proteins that have cytotoxic and anticancer properties. Some of these are known for their characteristic and marked immunomodulatory effects. These proteins are indicated as fungal immunomodulatory proteins (FIPs), whose mechanisms of action can be diverse [31,33]. Proteins also include lectins, which bind reversibly to mono- and oligosaccharides with high specificity, recognizing and interacting with various carbohydrates and proteoglycans on the cell surface. They are involved in many biological activities, such as innate immunity and cell-to-cell interaction, and their immunomodulatory mechanism varies depending on the origin of each compound. They also have immunomodulatory, antitumor, and antiproliferative properties [31,35]. 

Other compounds that play a pivotal role in the bioactivities of mushrooms are terpenes, quinones, triacylglycerols, isoflavones, catechols, and steroids. Other fungal metabolites showing interesting biological activities are phenolic compounds, antioxidants with different mechanisms of action (oxygen scavenging, metal inactivation, free radical inhibition, peroxidase decomposition), laccases (copper-containing oxidases), and fatty acids [36].

**Figure 2:**
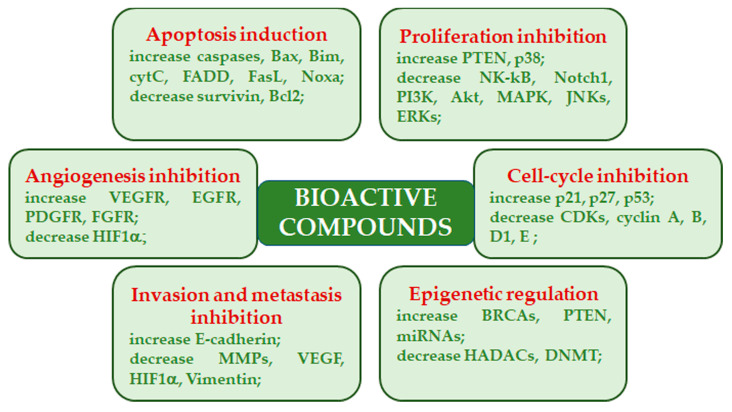
Some of the mechanisms related to the in vitro antineoplastic activity of bioactive mushroom compounds in BC.

In the past, high molecular weight compounds (i.e., polysaccharides and glycoproteins) were believed to exert their antitumor activity through the immune response activation, while low molecular weight compounds were believed to directly regulate signal transduction pathways linked to cancer development, progression, and survival. However, evidence has been reported indicating a direct action of certain polysaccharides on tumor cells [23]. Mushroom-derived polysaccharides exhibit potent antitumor activity against several kinds of metastatic cells. Moreover, they show increased activity when used in conjunction with chemotherapy. Mechanistically, antitumor activity is facilitated through a thymus-dependent immune mechanism, which necessitates an intact T-cell component. Polysaccharide class components mainly trigger cytotoxic macrophages, natural killer cells, dendritic cells, monocytes, neutrophils, and chemical messengers that activate complementary and acute phase responses. In addition, these polysaccharides act as multi-cytokine inducers, capable of stimulating gene expression of many immunomodulating cytokines and their receptors [37,38]. Various mushroom species, belonging to the genuses *Agaricus*, *Albatrellus, Antrodia*, *Calvatia*, *Clitocybe*, *Cordyceps*, *Flammulina*, *Fomes, Funlia*, *Ganoderma*, *Inocybe*, *Inonotus*, *Lactarius*, *Phellinus*, *Pleurotus*, *Russula*, *Schizophyllum*, *Suillus*, *Trametes*, and *Xerocomus* produce compounds with inhibitory activity on cancers. Besides the immunomodulatory effects, these compounds exhibit their anticancer effects by acting as angiogenesis inhibitors, antimitotic agents, mitotic kinase inhibitors, reactive oxygen species inducers, and topoisomerase inhibitors [31,39] (Figure 2).

## 3. Edible/Medicinal Mushrooms in Breast Cancer

As already mentioned, several edible/medicinal mushrooms have been studied for their potential antineoplastic effects on BC, which is still one of the main causes of death in the world. The inclusion of mushrooms in the diet has been shown to be protective against cancer [40]. Furthermore, dietary consumption of mushrooms has been associated with a diminished risk of BC [41,42].

**Figure 3:**
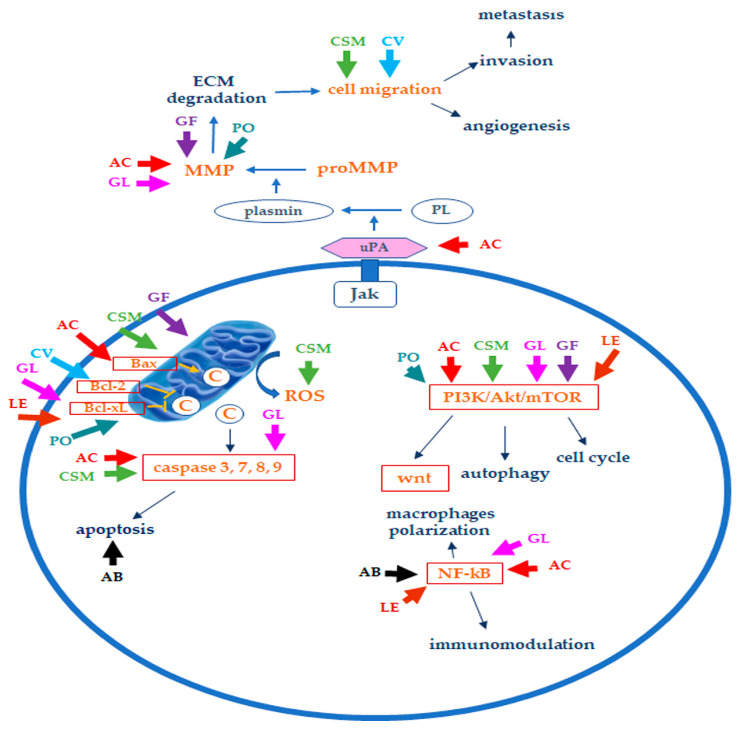
Simplified scheme of the mechanisms involved in the effects of the considered mushrooms on breast cancer (*Agaricus bisporus*, AB; *Antrodia cinnamomea*, AC; *Cordyceps sinensis* and *Cordyceps militaris*, CSM; *Coriolus versicolor*, CV; *Ganoderma lucidum*, GL; *Grifola frondosa*, GF; *Lentinula edodes*, LE; *Pleurotus ostreatus*, PO).

Here, we have considered some of the edible/medicinal mushrooms with inhibitory action on BC and the possible mechanisms responsible for their effects. A summary of the principal bioactive components contained in the considered mushrooms, along with their possible anticancer mechanisms, is reported in Table 1, while Figure 3 reports a simplified scheme of the mechanisms involved in the anticancer effects of the considered mushrooms on TNBC. Table in Section 4.2 summarizes the in vitro and in vivo evidence of their effects on BC reported in this review.

### 3.1. Agaricus bisporus (J.E. Lange) Imbach

*Agaricus bisporus* is an edible basidiomycete mushroom belonging to the family of *Agaricaceae*, native to grasslands in Eurasia and North America. *A. bisporus* is one of the most widely consumed mushrooms worldwide, and indeed it is cultivated in more than seventy countries. The immature state is characterized by a closed cap and can present two different colors, white and brown. The white one is also known as the common mushroom, white mushroom, button mushroom, or champignon, while the brown one is known as the Swiss brown mushroom, Roman brown mushroom, Italian brown mushroom, cremini/crimini mushroom, or chestnut mushroom. Regarding the mature state, it is brown with an open cap and is commonly sold under the names of portobello, portabella, or portobella.

*A. bisporus* is a precious source of amino acids, unsaturated fatty acids (including linoleic and linolenic acids), vitamins B, vitamin C, sterols, phenolic and indole compounds, beta-glucans, ergosterol, ergothioneine, vitamin D, and flavonoids, which are present in different concentrations depending on the cooking method, and on the exposure to UV light. For example, the ergocalciferol (vitamin D_2_) content increases substantially after exposure to UVC. As a matter of fact, unlike plants, the mushrooms’ cell wall possesses a high content of ergosterol, which plays a similar role to cholesterol in the animal cell wall. Upon exposure to UV light, ergosterol is transformed into pre-vitamin D_2_, which subsequently undergoes a temperature-dependent reaction that leads to ergocalciferol [31,43,44].

Thanks to the high content of nutrients, the fruiting bodies of *A. bisporus* have important anti-oxidant, anti-bacterial, anti-inflammatory, anti-tumoral, and immunomodulatory activities [45]. 

Conjugated linoleic acid is one of the bio-active components of *A. bisporus*, with a significant role in chemoprevention and inhibition of carcinogenesis. As a matter of fact, *A. bisporus* possesses interesting potential in protecting against some cancers, including breast cancer [31,46]. In addition, conjugated linoleic acid from white button mushroom extracts was able to inhibit testosterone-induced cell proliferation in MCF7 cells without affecting non-tumorigenic MCF-10A cells. Moreover, the extract was able to suppress tumor growth in nude mice bearing MCF-7 xenografts [47].

Several studies have evaluated the effects of polysaccharides, mainly β-glucans, from *A. bisporus* and their immunomodulatory effects. In this context, evidence has been reported about the potentiality of linear β-(1→6)-d-glucan (B16) isolated from *A. bisporus* in inducing macrophage polarization towards the M1 phenotype, which presents anti-tumoral activities with the production of pro-inflammatory markers (IL-1 β, TNF-α, CoX-2). Unfortunately, MDA-MB-231 BC cells were insensitive to B16; however, combined treatment with B16 and doxorubicin resulted in a synergic effect, providing an increase in M1 polarization and an improvement of BC cell sensitivity to doxorubicin [48]. Previously reported data concerning the effects of two distinct polysaccharide fractions (ABP-1 and ABP-2) extracted from *A. bisporus* on murine macrophages demonstrated the induction of nitric oxide, Interlukin-6, and Tumor Necrosis Factor-α (TNF-α) production, in part through the activation of Nuclear factor-kB. Moreover, both fractions were able to inhibit human BC MCF7 cell growth, probably also due to their activity on macrophages [49].

Lectins present in *Agaricales*, as well as in other medicinal fungi, showed therapeutic properties against many cancers in both animal and clinical studies. However, the specific anti-cancer mechanisms of lectins have not yet been fully elucidated. What is known is that the preferential binding of lectins to sugars present on cancer cell membranes causes cytotoxicity and apoptosis. Lectins may also alter the production of interleukins, acting at the level of the immune system. Furthermore, they can bind to ribosomes, thus modulating the proteome of the cell and inhibiting protein synthesis [50]. Moreover, the antiproliferative effect of lectins present in *A. bisporus* is likely to be a consequence of the inhibition of protein uptake into the nucleus, following the lectin trafficking to the nuclear periphery, where it blocks sequence-dependent protein import [33].

Recently, a lectin-like protein from *A. bisporus* has been shown to recognize and bind mannose on MCF7 cellular membranes, inducing cell death at high concentrations (100 µg/mL) and cell growth arrest at lower concentrations [51]. 

Interestingly, for their ability to bind glycoproteins and glycolipids, lectins from mushrooms could be a potential source for detection of glyco-markers, which are aberrantly expressed on the cancer cell surface (e.g., T/Tn antigens, Lewis/Lewis X). This could have a positive impact on early detection and better prognosis, as well as on follow-up and responsivity to cancer therapy progression and evolution [52].

### 3.2. Antrodia cinnamomea T.T. Chang and W.N. Chou

*Antrodia cinnamomea* (Syn. *Antrodia camphorata*) belongs to the family of *Polyporaceae*. Its current name is *Taiwanofungus camphoratus* (M. Zang and C.H. Su) Sheng H. Wu, Z.H. Yu, Y.C. Dai, and C.H. Su). *A. cinnamomea* is a precious and rare edible and medicinal mushroom that grows slowly and exclusively on decaying empty trunks of the *Cinnamomum kanehirae* Hayata (*Lauraceae*), an endangered aromatic tree endemic to Taiwan. 

Due to the rarity of this product, and the high market demand, the fruiting bodies of this mushroom are very expensive, which recently encouraged the development of novel cultivation techniques to provide a steady supply at reasonable prices [53,54]. *A. cinnamomea* is also extremely valued, as it contains bioactive compounds with potential anti-cancer, antioxidant, anti-inflammatory, anti-hypertensive, anti-hyperlipidaemic, hepatoprotective, and immunomodulatory activities, as well as regulating properties of the gut microbiota. In the past, this mushroom was used by native populations to treat liver diseases, particularly the disorders caused by alcohol intoxication. Nowadays, it is used as a chemopreventive agent in folk medicine [55].

Several bioactive compounds from *A. cinnamomea* have been identified, including terpenoids, polysaccharides, lignans, glycoproteins, benzene derivatives, ubiquinone derivatives, and maleic and succinic acid derivatives [56]. In particular, ergostanes, found only in fruiting bodies, and lanostane, present in both fruiting bodies and cultured mycelia, are among the most interesting terpenoids in *A. cinnamomea*, and Anticin A, which comprehends different bioactive mycoconstituents, has shown important anti-inflammatory and anti-tumoral effects [31,56].

As regards BC, Anticin-A demonstrated the ability to arrest the epithelial-to-mesenchymal transition (EMT) in human estrogen receptor-positive MCF7 and triple-negative MDA-MB-231 cells, acting through the upregulation of the epithelial markers E-cadherin and occludin, and through the concomitant downregulation of the mesenchymal markers N-cadherin and vimentin. At the molecular level, Anticin-A appears to act through the p53 pathway by inducing miR-200, a repressor of ZEB1, known to be an important actor of EMT via downregulation of E-cadherin expression [57,58]. Furthermore, Antrocin C, belonging to the group of maleic acid and succinic acid derivatives, has been demonstrated to reduce the migratory capacity of human BC cells in vitro. In addition to the upregulation of epithelial markers (E-cadherin and occludin) and the downregulation of mesenchymal markers (N-cadherin and vimentin), Antrocin C was able to significantly inhibit TGF-β1-induced migration and invasion of MCF7 cells through the suppression of the β-catenin transcription factor, ultimately leading to a reduction in the expression of matrix-metalloproteinases MMP-2 and MMP-9. Moreover, Antrocin C was able to reverse EMT in MCF7 cells, probably acting on Smad2/3, a transcriptional factor belonging to the TGF-β pathway. This same phytochemical has also shown an interesting activity on metastatic MDA-MB-231 cells, as it inhibited both Akt/mTOR and NF-κB pathways, thus acting as a promising dual inhibitor of one of the major oncogenic driver pathways in many cancers [59]. Furthermore, Antrocin induced apoptosis by downregulating Bcl-2, Bcl-xL, and survivin expression, as well as by upregulating cytosolic cytochrome C and Bax [58].

Several in vitro studies demonstrated potent anti-proliferative, anti-metastatic, and cytotoxic activities of *A. cinnamomea* fermented culture broth (FCB) on tumor cells. For example, it has been shown that FCB promotes cell cycle arrest and apoptosis and also possesses anti-metastatic activity on the highly metastatic BC cell line MDA-MB231 [60]. Furthermore, another in vitro study on MCF7 cells showed an FCB dose- and time-dependent induction of apoptosis, demonstrated by loss of cell viability, chromatin condensation, internucleosomal DNA fragmentation, sub-G1 phase accumulation, the release of cytochrome C in the cytosol, activation of caspase3, and degradation of poly(ADP-ribose) polymerase (PARP) [61]. In addition, analysis of ROS species generation showed a dose-dependent activity of FCB in MCF7 cells.

The effects of the ethanolic fruiting bodies fraction of *A. cinnamomea*, containing antcin K, antcin C, antcin B, methyl antcinate B, eburicoic acid, and dehydroeburicoic acid, have also been evaluated in some studies, showing a potent growth inhibitory activity associated with apoptosis, increased by the concomitant administration of tamoxifen, on an acquired tamoxifen-resistant MCF7 cell line. This effect seemed to be due to the inhibition of mRNA expression of skp2 (S-phase kinase-associated protein 2), whose overexpression in BC is synonymous with poor prognosis [62]. The ethanolic extract also showed antiproliferative activity in human hormonal receptor-expressive T47D breast cancer cells in vitro and in vivo [31].

The ethanolic extract of dish-cultured *A. cinnamomea* (EEAC) on endoplasmic reticulum (ER) stress and histone deacetylate (HDAC) inhibition in breast cancer cells (T47D, MCF7, and MDA-MB-231) was also studied; both the induction of cell cycle arrest in G1 and the inhibition of HDACs were observed. Moreover, the presence of the autophagic marker LC3-II of the p62 protein and of the FOXO1 transcription factor was detected, thus suggesting that the autophagic process is a central mechanism of inhibition of proliferation of T47D cells upon exposure to EEAC [53,58].

The potential anti-tumor effect of EEAC has also been confirmed in vivo in nude mice. Reduction of tumor size without observation of significant side effects was documented, suggesting a possible role for EEAC as an anti-cancer agent, particularly in the field of HDAC inhibitors [53].

### 3.3. Cordyceps sinensis (Berk.) Sacc. and Cordyceps militaris (L.) Fr.

Widely used in traditional Chinese medicine, medicinal mushrooms of the *Cordyceps* genus have shown beneficial effects on human health thanks to their immune stimulatory, neuroprotective, antimicrobial, anti-inflammatory, and anticancer activities. *C. sinensis* (current name is *Ophiocordyceps sinensis* (Berk.) G.H. Sung, J.M. Sung, Hywel-Jones, and Spatafora) and *C. militaris* are the most known and studied among the more than 680 species belonging to the *Cordyceps* genus [63,64,65]. They are entomopathogenic fungi; namely, they parasitize insects by attacking the underground pupae and larvae of lepidoptera, ultimately producing a fruiting body that can be used as a medicinal remedy [66]. *C. sinensis* is also known in China as ‘Dong Chong Xia Cao’, which means “winter worm summer grass”, and “caterpillar fungus”. It is native to the Tibetan Plateau, Bhutan, Nepal, and the northeastern regions of India and grows optimally at 3500–5000 m above sea level [64]. *C. militaris* is typically used in China and East Asia as a tonic [67] and is widely used as a crude preparation due to its multiple pharmacological properties [68].

Several active compounds are present in *Cordyceps* mushrooms, such as ergosterol, mannitol, and modified nucleosides [69], but the major functional component with medicinal properties shared by both the aforementioned fungi is cordycepin, whose anticancer effects on various tumors, including BC, have been reported [63]. These antitumoral properties are attributed to structural similarity with cellular nucleosides and adenosine: cordycepin, lacking oxygen at the 3′-position of its ribose moiety, acts like a nucleoside analog and inhibits the polyadenylation of the mRNA of cancer cells [65]. 

Lee et al. demonstrated that both cordycepin and ethanol extracts of *C. militaris* suppressed MCF7 cell proliferation in a concentration-dependent fashion. Specifically, the authors showed that exposure to *C. militaris* (100 μg/mL) and cordycepin (25 μM, 50 μM) induced apoptotic cell death. In particular, exposure to cordycepin induced apoptosis through mitochondria-mediated intrinsic apoptotic pathways by promoting expression and translocation of Bax to mitochondria, releasing cytochrome C, activating caspase-9, and activating extrinsic apoptotic pathways through the activation of caspase-8 [65]. In vitro experiments demonstrated that Cordycepin extracts inhibited growth, migration, and invasion of the human BT549 and mouse 4T1 TNBC cell lines, mainly through the reduction of proteins such as TWIST1, SLUG, SNAIL1, and ZEB1 involved in EMT. Among its various actions, Cordycepin also downregulates N-cadherin, triggering the overexpression of E-cadherin, thus inhibiting the migratory behavior of BC cells [63]. 

Cordycepin extracts from *C. sinensis* have also shown cytotoxic activity on TNBC MDA-MB-231 and MCF7 human BC cells. This effect was mainly due to lactate dehydrogenase cellular release, ROS production, and disruption of mitochondrial function. In addition, downregulation of the antiapoptotic Bcl-2 protein and upregulation of proapoptotic protein levels were observed. Autophagy, DNA damage, and targeting of cancer stem cells also contributed to the Cordycepin tumor-suppressing effect [58].

The effects of cordycepin have also been confirmed in 4T1 mouse models, as 30 days of treatment significantly decreased experimental tumor weight and size and reduced the number of metastatic colonies in the lungs [63]. Consistently, in vivo studies have demonstrated that *C. sinensis* reduced BC metastases in 4T1 tumor-bearing mice [66]. 

Furthermore, an aqueous extract of *C. militaris* exerted anticancer action and prolonged the survival of nude mice bearing MCF7 xenografts.

Interestingly, dietary administration of JLM 0636, a preparation of cordycepin-enriched *C. militaris* characterized by a seven-fold increased cordycepin concentration, led to tumor growth arrest and prolongation of survival in C3H/He mice bearing FM3A BC xenografts, possibly due to an increment of interferon-γ (IFN-γ) expressing cytotoxic T cells [58]. 

Finally, in MCF7 xenografts, administration of *C. militaris* for 14 days was shown to boost the expression levels of cleaved PARP, cleaved caspase-3, cleaved caspase-8, and Bax, thus supporting the existing evidence about the antitumoral effects of this mushroom [67]. 

### 3.4. Coriolus versicolor (L.) Quél.

*Coriolus versicolor* (Syn. *Polyporus versicolor*) is commonly known as Y(T)unzhi in China, Kawaratake in Japan, or Turkey tail in the Western world, and also as *Trametes versicolor* (L.) Lloyd, which is its current name. It belongs to the *Polyporaceae* family [70]. It is common in Asia, North America, and Europe. Its medicinal value is known in Chinese traditional medicine and includes general health-promoting effects. In Asian regions, and particularly in China, *C. versicolor* has been used as a “magic herb” to promote good health, strength, and longevity [71,72]. Since 1987 in China and 1977 in Japan, *C*. *versicolor* extracts have been approved in integrated cancer therapy in combination with chemotherapy or radiotherapy [31,70].

*Coriolus versicolor* contains various bioactive substances, including two protein-bound polysaccharides (i.e., the polysaccharide peptide, PSP, and the glycoprotein PSK, krestin), terpenes, proteins, peptides, amino acids, purpurins, and others. However, PSP and PSK are the most studied mushroom bio-compounds and the most active biological components in the mushroom [70].

PSP possesses immunomodulating effects, mainly due to the ability to act on cytokine release, to increase the expression of cytokines and chemokines, such as tumor necrosis factor-α (TNF-α), interleukins, histamine, and prostaglandin E, to activate natural killer (NK) cells and to enhance dendritic and T cell infiltration into tumors. Furthermore, antitumor, anti-inflammatory, and antiviral effects have been reported, along with other physiological effects, such as liver-protecting, system-balancing, antiulcer, antiaging and learning, and memory-enhancing properties. Interestingly, *C. versicolor* was also shown to reduce adverse effects related to chemotherapy and radiotherapy treatments [71,73].

Krestin showed both direct and indirect cytotoxic effects on cancer cells in vitro [74,75,76]. Thus, PSP and PSK can strengthen the body’s natural immune response, therefore exerting anti-tumor effects; however, the underlying mechanism has not been fully understood. 

A number of studies investigated the anti-tumor and anti-metastatic effects of *C. versicolor* on BC cell lines [77,78,79]. In particular, it has been shown that *C. versicolor* extract suppressed the proliferation of T47D, MCF7, and MDA-MB231 cells with a magnitude similar to the anti-cancer drug mitomycin C. These effects are exerted through the induction of apoptotic cell death, the upregulation of p53, and the downregulation of Bcl-2 [78]. The effects of a *C. versicolor* aqueous extract were also evaluated in mouse mammary carcinoma 4T1 cells and a 4T1-tumor-bearing mouse model, showing significant inhibition of cell migration, invasion, and MMP9 enzyme activity and protein downregulation. Animal studies showed that a *C. versicolor* aqueous extract induced a decrease in tumor weight and lung metastases. Furthermore, treatment with *C. versicolor* aqueous extract resulted in significant immunomodulatory effects, which were reflected by increased IL-2, IL-6, IL-12, TNF-α, and IFN-γ production in the spleen lymphocytes of *C. versicolor*-treated tumor-bearing mice [80]. The effects of *C. versicolor* extracts in metastasis-promoting chronic inflammation in BC cell lines were also evaluated, and antimigratory and cytotoxic effects were observed [81]. 

Recently, it has been reported that protein-bound polysaccharides from *C. versicolor* induced stimulation of the TNF-α/TNFR1 pathway, leading to cytotoxicity via necroptosis activation in MCF7 cells [82].

### 3.5. Ganoderma lucidum (Curtis) P. Karst. (Reishi)

*Ganoderma lucidum*, also known in China and Korea as ‘Ling-zhi’ (meaning “spiritual power grass”) and in Japan as ‘Reishi’ or ‘Mannentake’ (10,000 years fungus), is one of the most widely used medicinal mushrooms in the world. 

*G. lucidum* grows worldwide in temperate and subtropical areas: it is common in Europe, America, Canada, Africa, India, China, Japan, Korea, and other Southeast Asian countries. The Chinese mushroom differs from the European *G. lucidum* s.str., and several studies have been conducted to distinguish the one from the other. Nowadays, the Asiatic species is called the ‘Ling-zhi’ fungus and refers to the species *G. lingzhi* Sheng H. Wu, Y. Cao, and Y.C. Dai [83]. The two species have different morphological characteristics and seem to contain different amounts of triterpenes [23,84,85,86].

Known since ancient times in traditional Chinese medicine as the “mushroom of immortality”, *G. lucidum* has been used to promote well-being and longevity and is recognized for its hypoglycemic, immunomodulatory, antihypertensive, anti-diabetic, antioxidant, antihyperlipidaemic, antimutagenic, antiaging, antimicrobial, and hepatoprotective pharmacological properties [31,87]. In addition, it has also been widely used as an adjuvant in the treatment of various types of cancer [88,89], including breast cancer [90,91,92]. The polysaccharides and secondary metabolites present in *G. lucidum* are thought to be responsible for these biological activities. In particular, secondary metabolites from *G. lucidum* include triterpene compounds such as ganoderic acids, ganodermic acid, ganodermic alcohols, lucidones, lucinedic acids, ergosterol, 5,6-dehydroergosterol, ergosterol peroxide (EP), and palmitic acid. They possess antitumor, antimetastatic, cytotoxic, and enzyme inhibitory properties, while polysaccharides, mainly α-1,3, β-1,3, and β-1,6-D-glucans and ganoderan, are characterized by strong antiangiogenic and immune system-strengthening properties [89,93]. Therefore, these two categories of molecules are primarily responsible for the anticancer properties of Reishi by suppressing cell proliferation, metastasis, and invasion and by promoting apoptosis, combined with its immunomodulating, immunostimulating, antioxidant, and anti-inflammatory activities [20]. 

More than 100 Reishi-based products are currently marketed, such as nutraceuticals, supplements, functional foods, mycopharmaceuticals, and cosmeceuticals. 

Concerning BC, many studies associated whole mushroom extract or individual bioactive compounds of *G. lucidum* with cell death induction or cell cycle arrest of several human BC cell lines. In particular, cytotoxic ity and pro-apoptotic effects of total triterpenes from *G. lucidum* were evaluated in both noninvasive, estrogen-dependent, and highly invasive, estrogen-independent human breast adenocarcinoma cell lines, showing apoptosis induction and cell cycle arrest through downregulation of cyclin D1, Bcl-2, Bcl-xL levels, and upregulation of Bax and caspase-9 levels [91,94,95]. In vivo experiments in Wistar rats were also performed to evaluate the anti-carcinogenicity of total triterpenes using dimethyl-benz-[a]-anthracene (DMBA) to induce mammary adenocarcinomas. The results showed a significant reduction in the incidence of mammary tumors. In addition, total triterpenes were also found to reduce the average number of tumors per animal and extend the tumor latency period [96]. 

Recently, the most abundant components of whole *G. lucidum* extracts have been tested on a panel of triple-negative BC cell lines, showing the greatest selective anti-cancer activity of EP on BC cells and no significant toxicity against normal breast and non-cancerous mammary epithelial cells [89]. Specifically, this study demonstrated that EP exerts anti-proliferative effects through G1 phase cell cycle arrest, apoptosis induction via caspase 3/7 activation, and PARP cleavage, concomitantly inhibiting the expression of total AKT1, AKT2, BCL-XL, Cyclin D1, and c-Myc in BC cells. In addition, EP generated reactive oxygen species, thus compromising cellular viability. Moreover, EP decreased migratory and invasive effects of cancer cells at lower dosages than those reported in the literature in BC cell lines [97]. To confirm the antimetastatic effect of *G. lucidum* extract, other studies showed that extracts of this mushroom inhibited the release of MMP2 and MMP9 in triple-negative BC cells and that in nude mice, oral administration of GLE can inhibit breast-to-lung cancer metastases through the downregulation of genes associated with invasive behavior [92,98]. Furthermore, other in vitro and in vivo studies highlighted a selective action of *G. lucidum* extracts and the commercial extract ReishiMax GLpTM (carpophore and cracked spores) on mice injected with inflammatory breast cancer (IBC) on the protein expression of E-cadherin, mammalian target of rapamycin (mTOR), human eukaryotic translation initiation factor 4G (eIF4G), and p70 ribosomal protein S6 kinase (p70S6K) as well as on the activity of extracellular regulated kinase (ERK 1/2), along with the reduction in tumor size and weight [91,99,100].

An active fraction containing fucose glycoprotein, isolated from *G. lucidum* (Ling-Zhi) extract (FFLZ), was shown to exert immunomodulating activities by stimulating the expression of inflammatory cytokines and antibody-mediated cytotoxicity in cancer cells [101,102]. In BC models, FFLZ reduced tumor size and suppressed metastasis in vivo through the down-regulation of TGFR and downstream signaling pathways, including the phosphorylation of Smad2/3 and the expression of Smad4. Furthermore, FFLZ inhibited breast cancer cell migration and altered the epithelial-to-mesenchymal transition phenotype [103]. 

Recently, it was shown that a crude polysaccharide isolated from the fusion of *G. lucidum* and *Polyporus umbellatus* mycelia (namely, Khz) decreased the proliferation and induced the apoptosis on MCF-7 cells through intracellular [Ca^2+^] increase and apoptotic induction mediated by caspase-7, -8, and -9 [104,105]. 

Recently, an extract derived from the sporoderm-breaking spores of *G. lucidum* (ESG) has been shown to suppress 4T1 tumor growth in vivo rather than in vitro. In this context, ESG could significantly increase both the cytotoxic T cell (Tc) population and the ratio of Tc to helper T cells (Th) in the peripheral blood of tumor-bearing mice; a similar promotion of Tc was also found in tumor-infiltrating lymphocytes. Moreover, ESG markedly downregulated the two immune checkpoints, programmed cell death protein-1 (PD-1, in the spleen) and cytotoxic T lymphocyte antigen-4 (CTLA-4, in the tumor), suggesting that ESG could effectively restore the T cell paradigm by recovering the exhaustion status through the suppression of the co-inhibitory checkpoints [106]. Finally, the effects of *G. lucidum* fruiting bodies ethanolic extract (GLEet) on the expression of xenobiotic-targeting enzymes, on the oxidant–antioxidant and hormonal status of 7,12-dimethyl-benz[a]anthracene (DMBA)-induced experimental breast cancer was investigated in female Sprague Dawley rats. Oral administration of GLEet to tumor-bearing animals significantly diminished the levels of lipid peroxidation, thereby enhancing nonenzymatic antioxidant levels, and also positively regulated estrogen receptor hormone levels to near normal when compared with DMBA-treated rats. Moreover, it also positively modulated xenobiotic metabolizing enzymes [107]. 

Thus, all the reported evidence suggests that *Ganoderma lucidum* and its bioactive compounds are capable of inducing cytotoxicity, antiproliferative effects, proapoptotic processes, and cell cycle arrest as part of its anti-BC properties. In addition, the dietary administration of *G. lucidum* may be efficiently used as a chemopreventive agent against mammary carcinogenesis.

### 3.6. Grifola frondosa (Dicks.) Gray (Maitake) 

*G. frondosa*, belonging to the order of *Polyporales*, has a long history as a medicinal mushroom. It has different appellatives, according to the reference country—in the USA and Canada, it is commonly known as “sheep’s head”, ”king of mushrooms”, ”hen-of-the-woods”, and “cloud mushroom”; in Japan, it is termed ‘Maitake’, whose meaning is “dancing mushroom”. The reason for this peculiar nickname is possibly due to the singular morphology of the mushroom itself, characterized by petaloid basidiomata that extend from a common, thick, and whitish stem. It usually grows in clusters at the base of broadleaf trees, preferentially near oaks, but also on other deciduous trees such as beech, chestnut, elm, and maple. The ideal habitats for its growth are northern temperate forests in North America, China, and Japan; it is uncommon in Europe [23,108]. 

This mushroom is used as a culinary ingredient in Japan, where it is cultivated exclusively for this purpose. Only young Maitake are edible since the mushroom becomes harder as it ages. Along with its high nutritional and nutraceutical value, it has been demonstrated to possess a wide range of therapeutic effects, including immunomodulatory, antiviral, antidiabetic, antitumoral, and anti-inflammatory ones. 

The major bioactive components involved in these beneficial outcomes for health are the β-glucans, in particular, the so-called D-fraction, a β-glucan complex composed of about 30% protein. Specifically, the D-fraction is extracted from the fruit bodies of the mushroom and contains a unique structure composed of a 1,6 main chain having a greater degree of 1,3 branches and a 1,3 main chain with 1,6 branches. In addition to the D-fraction, Maitake contains many other bioactive substances, such as the X-fraction, Grifolan, the MZ-fraction, and MT-α-glucan. [108,109]. 

The *G. frondosa* D-fraction has been demonstrated to be the major component showing antitumoral effects, possibly due to its ability to tune immune responses and to have direct antiproliferative and cytotoxic effects in a variety of human cancer cells [23,108]. For this reason, the Maitake D-fraction (MDF) has been the focus of multiple investigations in cancer research, including breast cancer. In vitro studies on hormone-dependent MCF7 cells demonstrated that MDF promotes the release of cytochrome C from mitochondria, fostering cell dysfunction and apoptosis, whereas studies on TNBC MDA-MB231 cells indicate that it alters the expression of genes involved in cell growth, proliferation, and progression. Moreover, MDF is involved in the regulation of both migratory and metastatic processes by upregulating E-cadherin protein levels and promoting cell-substrate adhesion, as well as by downregulating cell motility through the remodeling of the actin cytoskeleton and by inhibition of MMP2 and MMP9, linked to invasive behavior. Furthermore, it has been established that MDF decreases cell viability by affecting the localization of β-catenin, which is often correlated with poor prognosis in breast cancer patients. Specifically, MDF decreases β-catenin expression in the cytoplasm and nucleus, stimulating membrane localization to facilitate the binding with E-cadherin, thus favoring its anti-metastatic activity [109]. 

Moreover, *G. frondosa* polysaccharides regulate cell viability by promoting the expression of pro-apoptotic proteins by altering the Bax/Bcl-2 ratio and dampening the pro-survival pathways coordinated by PI3K-Akt and ERK [58,109]. 

A number of studies confirmed that MDF also maintains its antitumoral properties in vivo. Interestingly, in mice harboring cancer xenografts, MDF induced immunostimulatory effects on macrophages, natural killer cells, and T cells [58]. Furthermore, studies carried out in a xenotransplanted murine model of TNBC human cells showed a decrease in distant lung metastases following MDF treatment [109]. In addition, the treatment affected the viability of hormone-independent LM3 cells in culture and the metastatic potential of cells in an LM3 syngeneic murine model. Finally, the Maitake D-Fraction Pro4x inhibited carcinogenesis, angiogenesis, and cancer invasiveness and prolonged survival in BALB/c mice bearing breast tumor xenografts [58].

The evidence shown in this section demonstrated that, differently from other medicinal mushroom extracts, the Maitake D-Fraction affects BC cell viability regardless of the hormone receptor and HER2 status of tumor cells. This compound could be useful to treat an array of different breast tumor subtypes, including hormone-dependent, hormone-independent, and triple-negative BC. The medicinal mushroom *G. frondosa* may become a potential therapeutic strategy for the management of BC [109].

### 3.7. Lentinula edodes (Berk.) Pegler (Shitake) 

*Lentinula edodes* represents the second most popular edible mushroom after *Agaricus bisporus* in the global market [110,111,112]. It is extensively consumed in Oriental and, more recently, Western cuisine. Besides its culinary value, this mushroom exerts positive effects on human health, such as antioxidant effects, due to the phenolic compounds and ergothioneine, hypocholesterolemic activity, due to ergosterol, β-glucans, and eritadenine, and antihypertensive effects, due to various peptides, including lenthionine [113]. However, Shitake has attracted clinical attention due to its immunomodulatory and antiviral capacities as well as its potent antitumor action on different types of cancer, including BC, mainly due to its glucan component lentinan [114].

In MCF7 and MDA-MB-231 BC cells, it has been observed that a peptide (latcripin-7A) extracted from *L. edodes* induced cell cycle arrest and decreased the mitochondrial membrane potential, leading to apoptotic cell death. Furthermore, in the same cell lines, the peptide significantly reduced migration and promoted autophagy without affecting the survival of MCF-10A normal breast cells [115]. The antiproliferative and proapoptotic effects of *L*. *edodes* mycelial and fruit body extracts were previously observed in MCF-7 [116]. Furthermore, in MCF7 and MDA-MB-453 cells, the ethyl acetate fraction of *L*. *edodes* induced apoptosis by increasing Bax and p21 levels and by decreasing cdk4 and cyclin D1, ultimately resulting in cell cycle arrest [117].

Interesting results have also been obtained in vivo, in nude mice in which the β-glucan from *L. edodes* (GLE) demonstrated a significant reduction of MCF7 tumor growth, possibly through the suppression of cell proliferation and promotion of apoptosis. In-depth analysis indicated that GLE inhibited multiple pathways, such as the ones modulated by PI3K/Akt/mTOR, NF-κB, ERK, ERα, caspase, and p53 [118]. 

### 3.8. Pleurotus ostreatus (Jacq.) P. Kumm.

*Pleurotus ostreatus*, also called oyster mushroom, belongs to the *Agaricaceae* family under the *Basidiomycetes* class [119,120]. The *Pleurotus* genus includes approximately 40 species of mushrooms, which are among the most cultivated and consumed in the world [121].

Phytochemicals present in *P. ostreatus* were screened, and the antioxidant, antibacterial, and anticancer activities of the ethanolic extract of *P. ostreatus* have been studied in-depth. In addition, the binding affinities of 32 biologically active compounds found in oyster mushrooms with EGFR, PR, and NF-κB proteins, which are overexpressed in breast cancer, were evaluated [122]. 

Hypocholesterolemic, free radical scavenging, antioxidant, antiatherogenic, anti-tumor, and immunomodulatory effects have been attributed to the bioactive compounds contained in *P. ostreatus*, such as α-glucans, β-glucans, lentanin, lipopolysaccharides, resveratrol, concanavalin A, the natural statin mevinolin, and many others [122]. Recently, some of these effects have been correlated to the antitumor activity of *P. ostreatus* [121]. A methanol extract prepared from the fruiting body of *P. ostreatus* inhibited MDA-MB-231 and MCF7 cell growth through the induction of cell cycle arrest at the G0/G1 phase, upregulation of the *p21*, *p53*, *p27*, and *p19* genes and downregulation of E2f transcription factor 1, PCNA, CDK4, CDK6, and Transcription factor DP-1 [123]. Consistently, *P. ostreatus* ethanolic extract inhibited growth and proliferation and induced oxidative stress and apoptotic cell death in MCF7 cells [121].

Recent in silico results provided evidence confirming that *P. ostreatus* may be a useful source of bioactive compounds responsible for its significant antioxidant, antibacterial, and anticancer properties [122].

The six linked glucans in *P. ostreatus* extracts potentiated the cytotoxic activity of natural killer cells against breast and lung cancer cells, which was associated with the induction of nitric oxide and interferon-γ through upregulation of *KIR2DL*. The cytotoxicity of the compound was augmented by interleukin-2 [58].

Oral administration of *P. ostreatus* ethanolic extract to rats bearing carcinogen-induced tumors led to a significant decrease in tumor volume and an increased body weight without any alterations in food and water intake or other behavioral patterns of the animals. Ethanolic extracts of *P. ostreatus* also downregulated estrogen and progesterone receptors, probably due to the presence of ergosterol, which most likely acted as an anti-estrogen block in receptor-mediated pathways. COX2 expression was also inhibited, resulting in a prostaglandin cascade and hence tumor suppression [124]. Lovastatin/mevinolin, present in the *P. ostreatus* ethanolic extract, inhibited angiogenesis and metastasis through the inhibition of MMP-2 and MMP-9 expression in the 4T1 metastatic breast cancer cell line [121]. 

*P. ostreatus* polysaccharides downregulated the expression of VEGF, resulting in the suppression of angiogenesis in MCF7 cells. In addition, they also induced apoptosis by increasing caspase-9, caspase-3, Bax, and phospho-JNK expression and by reducing mitochondrial membrane potentials. In rats carrying carcinogen-induced breast cancer, supplementation of the β-Glucan derived from *P. ostreatus* resulted in low tumor incidence, decreased tumor volume, and reduction in the total number of tumor nodules [121]. Furthermore, *P. ostreatus* extracts induced apoptosis and significantly reduced colony forming ability, cell viability, and tumor spheroid size of MCF7 cells. The increase in caspase3/7 activity, the upregulation of p53 and Bax, and the downregulation of Bcl2 raised the Bax/Bcl2 ratio, thus showing that apoptosis was mediated by the intrinsic pathway and by an alteration in the balance of pro-apoptotic and antiapoptotic genes [125]. 

Despite the promising results observed, little is still known about the detailed signaling cascade induced by *P. ostreatus* extracts in BC cells.

**Table 1:** Summary of the main bioactive constituents and of the in vitro and in vivo (in animal models) effects against breast cancer of the considered mushrooms and mechanisms involved in these effects.

Species	Main Bioactive Constituents	Mechanisms
***Agaricus bisporum***	Polysaccharides (ABP-1 and ABP-2 fractions), in particular, β-glucans (β-(1→6)-d-glucan, B16), lectins, amino acids, unsaturated fatty acids (linoleic and linolenic acids), vitamin B, vitamin C, sterols, phenolic and indole compounds, ergosterol, flavonoids, ergocalciferol, ergosterol	Inhibition of cell proliferation, suppression of tumor growth in nude mice xenografts; induction of macrophages polarization towards M1 phenotype and production of Il-6, IL-1 β, TNF-α, CoX-2; induction of nitric oxide, activation of NF-κB and cell growth inhibition, probably due to the activity on macrophages; inhibition of proteins synthesis; lectins induce cytotoxicity, apoptosis, and immune system modulation [33,47,48,49,50,51]
***Antrodia cinnamomea***	Polysaccharides, terpenoids (ergostane, lanostane), lignans, glycoproteins, benzene derivatives, ubiquinone derivatives, maleic and succinil acids derivatives, Anticin A, Antrocin C, Antcin K, antcin C, antcin B	Induction of apoptosis, suppression of mRNA expression of S-phase kinase-associated protein 2 (skp2); decrease of urokinase plasminogen activator (uPA) activity, uPA receptor (uPAR), vascular endothelial growth factor (VEGF), and MMP-9 and MMP-2; inhibition of TGF-β1-induced migration arrest epithelial to mesenchymal transition (EMT); suppression of the ERK1/2, p38, and JNK1/2 phosphorylation; inhibition of Akt/mTOR and NF-κB pathways; apoptosis induction, cell cycle arrest, antimetastatic effect, dysfunction of mitochondrial caspase-3/-9 activation, cytochrome c release, degradation of PARP, and Bcl2/Bax dysregulation; HDAC inhibition, autophagy induction (LC3-II, p62, and FOX1 increase) [31,53,57,58,59,60,61,62,126]; proliferation inhibition related to the arrest of cells at the G1 phase and induction of autophagy; stress of the endoplasmic reticulum; reduction of tumor size [62]
***Cordyceps sinensis and Cordyceps militaris***	Cordycepin (3-deoxyadenosine), ergosterol, mannitol, modifies nucleosides	Induction of apoptosis by promoting expression and translocation of Bax to mitochondria and decreasing Bcl2 levels by releasing cytochrome C, activating p53, caspase-9, caspase 3, caspase-8; inhibition of cell growth, migration, and invasion, through reduction of the EMT (TWIST1, SLUG, SNAIL1, ZEB reduction, N-cadherin downregulation, E-cadherin upregulation); inhibition of migration; antiproliferative activity through induction of apoptotic cell death; LDH release, PARP increase, ROS production, inhibition of AKT activation and PI3K/Akt; increased level of Cu/Zn superoxide dismutase in cancer cells; induction of autophagy, DNA damage, and targeting of cancer stem cells [58,63,65]; decrease in tumor weight and size; reduction of the number of metastasis; increase survival; increased expression levels of cleaved PARP, cleaved caspase-3, cleaved caspase-8, and Bax [63,66,67]
***Coriolus versicolor***	Protein-bound polysaccharides (polysaccharide peptide, PSP, and glycoprotein PSK, Krestin), terpenes, proteins, peptides, amino acids, purpurins	Suppression of cell proliferation through apoptotic cell death induction, upregulation of p53, and downregulation of Bcl-2; NK cell activation, p53, and Bcl-2 downregulation; inhibition of migration (MMP9 activity and protein levels downregulation); cytotoxicity via necroptosis activated through the TNF-α/TNFR1 pathway stimulation [77,78,79]; suppression of cancer cell proliferation, reduction of tumor weight and antimetastatic effect, simultaneously protecting bones against breast cancer-induced osteolysis; migration and invasion inhibition; immunomodulatory (increase IL-2, 6, 12 TNF-α, INF-γ, histamine, prostaglandin E) and antimigratory effects [80,81,82,126]
***Ganoderma lucidum***	Polysaccharides (α-1,3, β-1,3 and β-1,6-D-glucans, ganoderan), triterpenes, ganoderic acids, ganodermic acid, ganodermic alcohols, lucidones, lucinedic acid, ergosterol, 5,6-dehydroergosterol, ergosterol peroxide, and palmitic acid	Inhibitory effect against Akt phosphorylation on Ser473 and downregulation of Akt expression, inhibition of NF-κB, also related to estrogen receptors, cyclin D1, and subsequently cdk4 [126]; suppression of adhesion, migration, and invasion of cancer cells, down-regulation of oncogene c-myc expression and secretion of uPA and inhibition of MMP2 and MMP9 [92,98]; apoptosis induction through downregulation of cyclin F, Bcl-2, Bcl-xL and upregulation of Bax and caspase-9 levels [91,94,95]; G1 phase arrest, apoptosis induction via caspase 3/7 activation, and PARP cleavage [89]; inhibition of tumor growth and migration via inhibition of Wnt/β-catenin signaling; suppression of cancer cell growth through apoptosis induction via mitochondria-mediated pathway; effects on protein expression of E-cadherin, mammalian target of rapamycin (mTOR), human eukaryotic translation initiation factor 4G (eIF4G), and p70 ribosomal protein S6 kinase (p70S6K) and activity of extracellular regulated kinase (ERK 1/2), reduction in tumor size and weight; downregulation of immune checkpoints; effects on cancer stem cells [91,99,100,106,126,127]; reduction in incidence of mammary tumors [96]
***Grifola Frondosa***	β-glucans and α-glucan (D-fraction, X-fraction, Grifolan, MZ-fraction, and MT-α-glucan), proteins, carbohydrates, ergocalciferol, minerals	Apoptosis induction through the release of CytC from mitochondria, alterations in genes involved in cell proliferation and invasion; upregulation of E-cadherin protein levels, promotion of cell adhesion, downregulation of cell motility and MMP2 and MMP9; decrease in β-catenin levels; modulation of Bax/Bcl2 ratio, affecting the pro-survival pathways related to PI3K/Akt and ERK [58,109]; immunomodulatory effects on macrophages, NK and T cells; decrease in metastasis; inhibition of carcinogenesis, angiogenesis, and cancer invasiveness; prolonged survival [58,109]
***Lentinula edodes***	β-glucans (lentinan), phenolic compounds, ergothioneine, sterols (ergosterol), eritadenine, peptides (lenthionine)	Induction of apoptosis associated with mitochondrial membrane potential decrease and decreased cdk4 and cyclin D1 resulting in cell cycle arrest; increased p21, p53, and Bax levels; inhibition of migration, autophagy induction [115,116,117,126]; reduction in tumor growth through suppression of cell proliferation and apoptosis promotion; inhibition of multiple pathways (PI3K-Akt-mTOR, ERK, p53) [118]
***Pleurotus ostreatus***	α-glucans, β-glucans, lentanin, lipopolisaccharides, resveratrol, concavallin A, mevinolin, ergosterol	Cell growth inhibition related to cell cycle arrest at the G0/G1 phase, upregulation of the p21, p53, p27, and p19 genes and downregulation of E2f transcription factor 1, PCNA, CDK4, CDK6, and transcription factor DP-1; induction of oxidative stress and apoptotic cell death due to the upregulation of p53 and Bax, downregulation of Bcl2, and increase in caspase 3/7 activity; increased cytotoxic activity of natural killer cells; inhibition of angiogenesis and metastasis by the inhibition of MMP2 and MMP9 expression; downregulation of VEGF [58,121,124]; decrease in tumor volume and increased body weight; decrease in tumor incidence, volume, and metastasis [121,124,125]

## 4. Human Studies

To retrieve the available published evidence concerning studies performed in humans, electronic databases (PubMed, Embase, SCOPUS) were searched for articles published in English or other European languages, using broad search terms (e.g., for human studies: ((mushroom(MeSH Terms)) AND (breast cancer(MeSH Terms))); restricted to observational studies: ((mushroom(MeSH Terms)) AND (breast cancer(MeSH Terms))) AND (case control study(MeSH Terms)), or ((mushroom(MeSH Terms)) AND (breast cancer(MeSH Terms))) AND (cohort study(MeSH Terms)). Hand-searching was also performed in the reference lists of retrieved records. 

**Figure 4:**
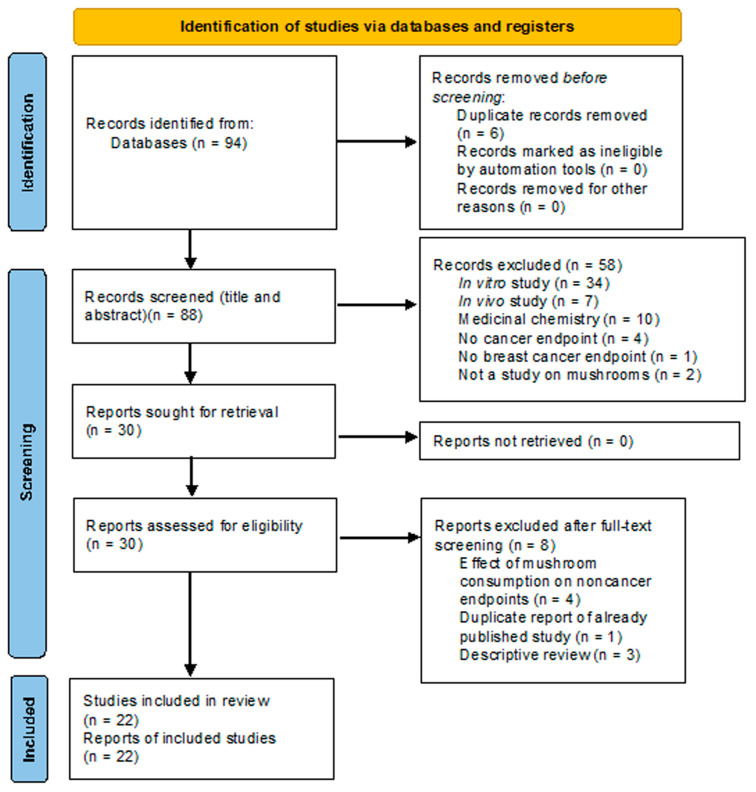
PRISMA flow chart of the process of record retrieval and screening, with reasons for exclusion.

A PRISMA flow chart [128] of the process of record retrieval and screening, with reasons for exclusion, is shown in Figure 4.

Eighty-eight deduplicated records were retrieved by database consultation and hand-searching. Fifty-eight and eight records were excluded after title/abstract and full-text screening, respectively (performed by two authors). Twenty-two articles reporting epidemiological and clinical studies were finally included in the review.

### 4.1. Studies on Dietary Consumption of Edible Mushrooms and Breast Cancer Risk

Long-term longitudinal studies performed on large populations point to a positive survival effect of dietary consumption of mushrooms. In the USA, a Cox proportional hazards regression analysis, performed on 15,546 participants in the frame of the Third National Health and Nutrition Examination Survey (NHANES III) over the average span of 19 years, showed that the hazard ratio (HR) for overall mortality was lower in subjects whose diet included regular consumption of mushrooms (HR adjusted for demographic factors, lifestyle, and overall diet quality, 0.84; 95% CI, 0.73 to 0.98). In addition, consumption of a daily portion of mushrooms in place of a daily serving of processed or red meat reduced the risk of all-cause mortality to 65% (HR, 0.65; 95% CI, 0.5 to 0.84). However, when cause-specific mortality was analyzed, the hazard ratio for ‘cancer’ (HR, 0.77; 95% CI, 0.5 to 1.19), as well as for other major causes of death, such as cardiovascular diseases or diabetes mellitus, did not reach statistical significance [41].

A 2014 dose-response meta-analysis of eight case-control and two cohort studies (6890 cancer cases) demonstrated that mushroom consumption may be inversely associated with the risk of breast cancer (BC). In particular, each one-gram-per-day increment in dietary intake of mushrooms appears to decrease the risk of BC by 3% (risk ratio (RR), 0.97; 95% CI, 0.96 to 0.98). The RR for postmenopausal women decreases to 0.94 (95% CI, 0.91 to 0.97) but loses statistical significance in premenopausal patients (RR, 0.96; 95% CI, 0.91 to 1.00) [42].

The data concerning breast cancer were confirmed by a more recent meta-analysis of 17 observational studies (6 cohort and 11 case-control studies), including 19,732 cancer cases. Analysis showed that mushroom consumption is associated with a significant reduction in the risk for ‘any cancer’ (RR, 0.66; 95% CI: 0.55 to 0.78). Interestingly, cancer subgroup analysis showed that the major contributor to the significance of the meta-analysis was BC. As a matter of fact, the relative risk for BC was 35% lower in mushroom consumers compared to controls (RR, 0.65; 95% CI, 0.52 to 0.81) [41], whereas risk ratios for prostate, ovarian, stomach, liver, and colorectal cancers were not statistically significant, thus suggesting a specific activity of mushrooms on BC. Alternatively, according to Ba and coworkers, issues concerning the sample size of this meta-analysis may account for nonsignificant results in non-breast cancers. The latter hypothesis appears to be supported by other cohort studies performed in Asia, the pooled Japanese Miyagi Cohort Study, Ohsaki Cohort Study, and a Korean study showing that regular mushroom consumption is associated with a significant reduction in the hazard for prostate cancer [129] and gastric cancer [130].

Mushrooms are part of the daily diet in East Asia, whereas, in Western countries or other continents, mushroom consumption is less frequent. A case-control study performed in China included 1009 pre- and post-menopausal BC patients, and 1009 matched healthy controls. The odds of getting BC decreased along with the increase in dietary mushroom consumption and reached statistical significance for daily intakes of at least 10 g of fresh mushrooms (odds ratio (OR) after adjusting for confounders, 0.36; 95% CI 0.25 to 0.51) or 4 g dried mushrooms (OR, 0.53; 95% CI, 0.38 to 0.73). In this study, a protective effect of mushrooms was observed in both premenopausal and postmenopausal women [131].

A similar Korean study compared 362 pre-and post-menopausal patients with 362 matched healthy controls. Daily mushroom intake was divided into quintiles. A significant inverse association between breast cancer and dietary mushroom consumption was found when the fifth quintile was compared to the lowest quintile (OR for daily intake adjusted for confounding factors, 0.55, 95% CI, 0.33 to 0.94) [132]. Subgroup analysis was performed by dividing cases and controls according to the menopausal status. Interestingly, a significant dose-dependent trend was confirmed in post-menopausal (*p* < 0.05 for trend) but not in pre-menopausal women (*p* > 0.05 for trend) in all tested multivariate-adjusted models. Nevertheless, significant protection was demonstrated in pre-menopausal women in one out of two adjusted models when dose quintiles were analyzed separately (e.g., OR at fifth dose quintile, 0.38; 95% CI, 0.19 to 0.77).

A subsequent Korean study by Shin and coworkers had a very similar design but appeared to be in partial disagreement with the findings outlined above. The study included 358 cases and 360 controls; mushroom intake doses were separated into quartiles. A significant, dose-related inverse association between BC and dietary mushroom consumption could be demonstrated by multivariate analysis in pre-menopausal (*p* < 0.05 for trend) but not in post-menopausal women (*p* > 0.05 for trend) [133]. The authors also investigated the relationship between the protective activity of mushrooms and the hormone status of breast tumors in affected patients. In the total population, including both pre- and post-menopausal women, a significant, dose-dependent inverse association between BC and dietary mushroom consumption was found in estrogen and progesterone receptor positive (ER+ PR+) patients (*p* < 0.001 for trend, multivariate analysis adjusted for confounding factors) but not in ER-/PR- patients (*p* > 0.05 for trend, multivariate analysis). Interestingly, significance was confirmed in ER+/PR+ pre-menopausal women but not in ER+/PR+ pre-menopausal subjects (*p* < 0.01 and *p* > 0.05 for trend, respectively, multivariate analysis). In summary, a high-dose intake of mushrooms is inversely associated with the odds of BC in pre-menopausal Korean women. In addition, mushroom consumption may play a more decisive role in patients with ER+/PR+ tumors. The aromatase inhibitory activity of certain mushrooms may be involved in the latter effect.

Whereas investigations performed in Asia yielded significant results, large population studies performed in Western countries (UK, USA) failed to demonstrate a link between dietary mushroom intake and decreased cancer risk.

The UK Women’s Cohort Study (n = 35,372 at baseline) did not find a significant inverse relation between mushroom consumption and BC hazard in the general population (HR, 0.98, 95% CI, 0.79 to 1.22). Lack of an effect was confirmed in both pre-menopausal (HR, 0.94; 95% CI, 0.51 to 1.75) and post-menopausal subgroups (HR, 1.03, 95% CI, 0.77 to 1.38) [40]. Similarly, a large prospective cohort study, including 68,327 women from the Nurses’ Health Study (USA, 1986–2012) and 44,644 men from the Health Professionals Follow-up Study (USA, 1986–2012), failed to demonstrate a decreased hazard for any cancer (HR, 1.02; 95% CI, 0.97 to 1.07) in subjects consuming five or more servings of mushrooms per week versus subjects who rarely consumed mushrooms (HR, 1.06; 95% CI, 0.98 to 1.14). In addition, no significant effects on site-specific or sex-specific cancers (e.g., for breast cancer, HR, 0.89, 95% CI, 0.77 to 1.04) were found [134].

The authors of both UK and USA studies concluded that the discrepancy between Asian and Western studies ought to be investigated. Genetic factors, ethnicity factors, the amount of mushroom consumed daily (higher in the East), the mushroom species consumed in the East but not in the West, the use of fresh mushrooms in the East versus processed mushrooms in the West, as well as cultural issues (cooking and preparation procedures, preserving the integrity of bioactive compounds) may be responsible for the differences between the results of studies performed in Eastern Asia and Western countries. In our opinion, the objective limitation of these studies lays in the positive effects attributed to the broad category of “mushrooms”. For the sake of advancement in the knowledge of the nutraceutic properties of mushrooms, studies should- be aimed at assessing the properties of specific mushroom species.

### 4.2. Effect of Fungal Extracts on Breast Cancer: Clinical Studies and Meta-Analyses

Table 2 summarizes the major findings from clinical studies focusing on treatment with medicinal mushrooms in BC patients.

Valadares et al. reported the results of a randomized, double-blind, placebo-controlled trial performed in 46 breast cancer patients (stages II and III) receiving a supplement of *Agaricus sylvaticus* (2.1 g daily) during 3/6 cycles of chemotherapy. The authors reported a protective effect of the mushroom supplement against various gastrointestinal adverse events caused by chemotherapy compared to placebo. In particular, two patients (15.3%) treated with *A. sylvaticus* reported nausea and vomiting, compared with 11 patients (84.62%) in the placebo group. Patients subjected to three cycles of chemotherapy at baseline reported poor appetite. The anorexant effect of chemotherapy was reported in 23.08% of patients in the placebo group and in 53.85% of patients treated with *A. sylvaticus*. After 3 months, 30.77% of patients in the placebo group still reported a reduction in appetite, whereas this effect was not reported in the cohort treated with *A. sylvaticus* [135].

Tsai and coworkers reported the results of a randomized, double-blind, placebo-controlled trial performed on 37 patients affected by advanced cancers, including TNM stage IV BC (n = 8). Breast cancer patients treated with adriamycin- or taxane-based chemotherapy were randomized to placebo or 20 mL daily of an oral aqueous extract of *Antrodia Cinnamomea* (AC) for 30 days [136]. No significant differences between treatment arms were found with respect to the primary (overall survival) or secondary endpoints (disease control rate, quality of life (QoL), adverse events). However, AC-treated patients showed significantly lower platelet counts. The authors did not mention BC and suggested that the latter effect may occur in patients with lung or gastric cancer. In addition, patients using AC experienced better quality of sleep compared to placebo (*p* = 0.04).

A phase I/II dose-escalating trial was performed in 34 BC patients to evaluate the safety, tolerability, and immunological effects of a liquid standardized extract of maitake administered at the daily doses of 0.2, 1, 3, 6, or 10 mg/kg for 3 weeks. The extract was well tolerated, and no dose-limiting toxicity was found. Interestingly, the immunological effects of maitake were documented. Blood analysis showed moderate increases of IL-10 and IL-2, as well as IFN-*γ* and TNF-*α* upon stimulation with various factors (LPS, PMA). Interestingly, efficacy followed a bell-shaped curve, peaking at intermediate doses (5–7 mg/kg) and decreasing at higher doses. The authors explain that whereas the immune stimulatory activity of IL-2 and IFN-*γ* may be functional in response to cancer, the anti-inflammatory role of IL-10 may not play a relevant role in the antitumor effect of maitake. Dose-dependent increases for CD4+ CD25+ T cells, CD3+ CD25+ T cells, CD45RA+ CD4+ cells, and CD45RO+ CD8+ cells were also reported [144]. 

Preliminary data from clinical trials suggest that *Coriolus versicolor* might become an adjunct agent to standard therapy for BC. A mushroom-herbal combination of Yunzhi (*Coriolus versicolor*) and Danshen (*Salvia miltiorrhiza*) was administered to 82 patients with BC. Patients received oral Yunzhi polysaccharopeptide (PSP, 50 mg/kg body weight daily) and Danshen (20 mg/kg body weight daily) for 6 months. Absolute counts of T-helper lymphocytes (CD4+) and B-lymphocytes, the ratio of T-helper (CD4+)/T suppressor cells, and the counts of cytotoxic lymphocytes (CD8+) were significantly elevated in patients receiving the Yunzhi/Danshen combination. Moreover, plasma soluble IL-2 receptor (sIL-2R) levels were significantly decreased [138]. These results indicate an overall improvement in the immunological profile of enrolled patients. Subsequent studies confirmed the immune-modulating activity of *Coriolus versicolor.* In particular, a phase 1 dose-escalating trial aimed at testing safety, tolerability, and initial biological effects of CV was performed on nine stage I, II, or III BC patients in the post-chemotherapy, post-radiotherapy setting. The freeze-dried mycelial powder of CV was administered to three groups of three patients at the daily doses of 3, 6, and 9 g. Treatment was well tolerated, although three out of nine adverse events (anxiety, heartburn, and chest pain) were attributed to the mushroom preparation. Interestingly, the two higher doses of the compound were associated with a faster recovery of lymphocytes, and higher CD8+ T cell and CD19+ B cell counts when compared to historical controls. In addition, a temporary increase in NK cell activity was recorded in the 6 g dosage group [137].

Interestingly, a 2012 metanalysis by Eliza and coworkers showed that *Coriolus versicolor* may be responsible for a 9% absolute reduction in 5-year mortality in patients affected by various kinds of cancers, with a number-needed-to-treat equal to 11. This applied to patients with breast, gastric, or colorectal cancer, but not to esophageal cancer and nasopharyngeal carcinoma [148].

The mushroom *Ganoderma lucidum* (GL) is usually consumed by BC survivors and has been tested in the frame of clinical trials focusing on breast, colon, gastrointestinal, and nasopharyngeal cancer, mainly due to its activity on the immune system [149,150,151]. 

A meta-analysis showed that patients treated with GL in addition to radiotherapy and chemotherapy were better responders to treatment when compared to chemotherapy/radiotherapy alone (RR 1.5; *p* = 0.02). GL also caused increases in CD3+, CD4+, and CD8+ cells, as well as modest increases in NK-cell activity [139]. 

Recently, a comprehensive randomized, double-blind trial investigated the changes in the immunological profiles of 120 lung or postoperative BC patients (triple-negative stage I–III) treated or not with 2000 mg of *G. lucidum* spore powder twice daily for 6 weeks [140]. The goal of the trial was to profile the T lymphocyte subsets and the cytokines associated with an increased likelihood of benefitting from the immune-stimulating effects of *G. lucidum*. Patients responding to GL showed higher levels of CD3+, CD4+, and CD3+/HLADR- and lower levels of CD4+, CD25+, Treg (CD4+/CD25+), and CD3+/HLADR+ cells compared to untreated subjects. IL-12 levels were significantly higher, and IL-10 levels were lower during treatment. Moreover, immunosuppressive factors such as COX2 and TGF-β1 had lower prevalence in treated patients. Interestingly, GL was a possible response modifier when albumin-to-globulin and neutrophil-to-lymphocyte ratios were used as prognostic predictors of overall survival and progression-free survival. The drug was well tolerated, and no serious adverse events were reported [140]. 

Alterations of cytokine profiles were also reported by Nidhal and coworkers, who performed an observational prospective study in 40 patients with advanced breast cancer treated with standard chemotherapy alone or combined with a GL preparation (one gram capsule twice daily for 12 weeks). In the GL group, IFNγ increased significantly, whereas TNF-*α* and IL-8 decreased significantly; no significant changes were observed in the chemotherapy-alone cohort [141].

A randomized, placebo-controlled trial investigated the effectiveness of a spore powder preparation of *G. lucidum* (1 g thrice daily for 4 weeks) in BC patients subjected to endocrine therapy. The endpoints were alterations in cytokine levels (TNF-α, IL-6), cancer-related fatigue, anxiety, depression, and quality of life. Serum TNF-α and IL-6 levels were significantly lower in the GL patient arm compared to placebo. Total and subdomain (fatigue, well-being) scores of the FACT-F (Functional Assessment of Cancer Therapy: Fatigue) test were significantly improved in the GL arm at the end of treatment, whereas no significant differences were found between baseline and at the end of 4 weeks in the placebo group. HADS (Hospital Anxiety and Depression Scale) scores of anxiety and depression after GL were lower than the scores before the intervention, but no significant changes were found in the placebo group. Consistently, the EORTC QLQ-C30 (European Organization for Research and Treatment of Cancer Core Quality of Life Questionnaire C30) ‘physical function’ and ‘global quality of life’ domains were significantly improved after the 4-week GL spore powder treatment. However, no significant changes were found in the ‘role functioning’ and ‘social functioning’ domains. Compared to patients in the placebo group, the ‘fatigue’, ‘sleep disturbance’, and ‘appetite loss’ domains were significantly improved in the GL treatment arm [142].

The effect of GL on the quality of life of breast cancer patients included in the Shanghai Breast Cancer Survival Study (SBCSS) was investigated by Bao et al. [143]. Of the 4149 participants who completed the 36-month survey, 58.8% and 36.2% reported *G. lucidum* use at the 6- and 36-month surveys, respectively. The use of GL was not significantly associated with improvements in QoL or psychological well-being at the end of a 36-month follow-up period. However, higher scores for social well-being and material well-being but lower scores for physical well-being were documented. In addition, patients using GL showed higher scores for self-image, social support, and interpersonal relationships but lower scores for sleep and energy, physical comfort, and eating functions.

In an early study, the *L. edodes* polysaccharide lentinan was safely administered to patients affected by advanced or recurrent breast cancer and seemed to improve the prognosis of the disease [152]. A group of ten BC patients was treated with cyclophosphamide, epirubicin, and 5-fluorouracil every 21 days for two cycles. An *L. edodes* mycelial extract was added to the therapeutic protocol during the second cycle of therapy. The *L. edodes* mycelial extract caused sustained natural killer cell activity and attenuated the drop in leukocyte counts [145]. 

Three patients undergoing postoperative adjuvant chemotherapy for BC underwent a first protocol of chemotherapy, followed by a second treatment phase consisting of chemotherapy to which an *L. edodes* mycelial extract was added. Increases in immunosuppressive acidic protein levels, NK cell activity, and quality of life of patients were documented after the second “add-on” phase of treatment. Administration of the *L. edodes* mycelial extract was well tolerated [146]. Twenty breast cancer patients received postoperative estrogen-alone hormone therapy for 4 weeks with no detectable alterations in the quality of life or cytokine levels. When an *L. edodes* mycelial extract was added to the hormone therapy in the subsequent 8 weeks, quality of life scores were significantly increased. In a subgroup of six subjects regarded as having decreased immunity, the IFNγ/L-10 ratio was significantly increased from 0.06 at Week 4 to 0.13 at Week 12, and the IFNγ production level was significantly increased from 17 at Week 4 to 46 at Week 12. In contrast, immune parameters were unchanged in patients who had an IFNγ/IL-10 ≥ 0.2 at Week 4 [147].

In conclusion, it is emerging that certain medicinal mushrooms appear to improve the immunological profile and alleviate the toxic and adverse effects of chemotherapy or radiotherapy (i) by ameliorating some aspects of the quality of life of patients, (ii) by accelerating the increase of B- and T-cell counts, (iii) by increasing the activity and counts of innate immune cells, and (iv) by modifying the cytokine profile in treated patients, and, in particular, by upregulating anticancer cytokines and/or downregulating cytokines involved in cancer progression or metastasis. 

## 5. Discussion

Edible/medicinal mushrooms, widely used in Asian countries for their nutritional and health properties, have recently gained popularity in Europe, as well. Specifically, several epidemiological studies indicated that mushroom intake can protect against cancer, in particular, gastrointestinal and breast cancer. Furthermore, mushrooms have been used as therapeutic agents for different types of cancer, including breast cancer [31,58,153].

The available evidence shows that mushrooms may contain compounds having the potential to be used in both the prevention and treatment of cancer, as well as in the stimulation and recovery of the immune function. The results reported so far, based on experiments with tumor cell lines or animal models, have partially clarified the molecular mechanisms involved in mushrooms’ anticancer activities. Although preclinical research has given a host of quality data concerning the efficacy of mushrooms’ bioactive compounds at the molecular and cellular levels, information about the translational potential of these compounds in clinical practice is still insufficient. Indeed, whereas very few phase-I/II studies provided data about the possible toxicity and initial therapeutic effect of certain compounds, data on many other mushroom-derived products are lacking. Dose-escalation trials assessing the maximum tolerated doses and the human pharmacokinetics of promising compounds are warranted to uncover the full potential of mushroom therapy.

In addition, most clinical studies, both randomized and observational, have been designed to investigate the short-term effects of intake of mushroom-derived compounds. Thus, information on the long-term effects, whether beneficial or detrimental, of therapy administered over extended periods of time is lacking.

Furthermore, most published studies include a small number of patients, and the statistical power of the produced evidence is sometimes questionable. The majority of published studies are observational, with a cohort or case-control design. The results of these investigations are certainly useful, though randomized, double-blinded, adequately powered controlled trials on sufficiently numerous populations of patients are warranted before any conclusive evidence is drawn. In our opinion, special attention should be devoted to investigating the effect of medicinal mushrooms such as *L. edodes*, *G. lucidum*, *G. frondosa*, and *C. versicolor* on natural killer cell activity in cancer patients.

Systematic reviews and meta-analyses of available clinical data included a mixture of randomized prospective and observational cohort studies. Such a strategy, unavoidable in the absence of a sufficient number of randomized clinical trials, would result in a low quality of the meta-analysis evidence according to the criteria fixed by the GRADE consortium [154]. Optimally designed meta-analyses, including only randomized controlled trials, will provide conclusive evidence about the full potential of medicinal mushrooms in cancer, thus paving the way to new forms of cancer therapy.

Further studies are also warranted to investigate the reasons underlying the diverging results of studies performed in Eastern Asian and Western countries [29]. As a matter of fact, results from the available clinical studies appear to be rather preliminary and sometimes give contrasting results, probably due to the lack of standardization in both methods of extraction and the schedule of treatment. 

Another issue to resolve is related to the procedures followed to prepare medicinal mushrooms, which are various and often not described in detail. Full disclosure of the criteria whereby certain compounds are extracted and prepared is warranted. In this respect, metabolomic engineering and technologies may be very useful in the optimization and standardization of preparations [155].

In agreement with data previously reported by other authors [29], updated and summarized in Table 3, few of the considered mushrooms seem to deserve further clinical investigation to confirm their in vivo preclinical activity and to better understand the mechanisms related to their effects on BC. Adequately powered controlled trials will greatly expand our knowledge concerning this interesting and promising source of new agents.

## Figures and Tables

**Table 2:** Summary of the major findings of clinical studies performed on medicinal mushrooms. (↑: increased; ↓: decreased; //: not available).

Species	Trial Design, Trial Identifier (When Available), Number of Patients (n)	Symptomatic Effects, Adverse Effects	Immune Cell Effects (vs. Controls)	Cytokine Level Effects (vs. Controls)	Other Effects	Reference
***Agaricus sylvaticus***	Randomized, placebo-controlled, double-blind trial, n = 46	↓ GI adverse effects and anorexia vs. placebo	//	//	//	[135]
***Antrodia cinnamomea***	Randomized, double-blind, placebo-controlled trial, clinicaltrials.gov identifier: NCT01287286, n = 37	↑ GI adverse effects vs. placebo	//	//	Overall survival, disease control rate, quality of life, adverse events: no differences between arms; platelet counts ↓ in AC arm; quality of sleep ↑ in AC arm	[136]
***Coriolus versicolor***	Phase I, dose-escalation trial, clinicaltrials.gov identifier: NCT00680667, n = 11	Severe anxiety (adverse effect)	↑ CD8+ T cells, ↑ CD19+ B cells; CD16+/56+ NK cell counts unchanged but activity ↑	//	//	[137]
	Cohort study, identifier not available, n = 82	//	↑ CD8+, ↑ CD4+, ↑ B-cells, ↑ T-helper/T suppressor cells ratio	↓ sIL-2R	//	[138]
***Ganoderma lucidum***	Meta-analysis, n = 153	//	↑ CD3+, ↑ CD4+, ↑ CD8+ cells; ↑ NK cell activity	//	//	[139]
	Randomized, double-blind trial, identifier not available, n = 69 (breast cancer)	No serious adverse effects	↑ CD3+, ↑ CD4+, ↑ CD3+/HLADR-cells; ↓ CD4+, ↓ CD25+, ↓ Treg (CD4+/CD25+), ↓ CD3+/HLADR+ cells	↑ IL-12, ↓ IL-10	//	[140]
	Prospective observational study, identifier not available, n = 40	//	//	↑ IFN-*γ*, ↓ IL-8, ↓ TNF-*α*	//	[141]
	Randomized controlled study, identifier not available, n = 48	Improvement of fatigue, anxiety, depression, QoL vs. controls; social functioning unchanged	//	↓ IL-6, ↓ TNF-*α*	//	[142]
	Population observational study, identifier not available, n = 4149	Improvement of QoL and physical and psychological well-being	//	//	//	[143]
***Grifola frondosa***	Phase I/II trial, identifier not available, n = 34	No serious adverse effects	↑ CD4+/CD25+ T cells, ↑ CD3+/CD25+ T cells, ↑ CD45RA+/CD4+ cells, ↑ CD45RO+/CD8+ cells	↑ IL-10, ↑ IL-2 and, ↑ IFN-*γ*, ↑ TNF-*α*	//	[144]
***Lentinula edodes***	Case series, add-on to chemotherapy, identifier not available, n = 10	//	Addition of LE to adjuvant chemotherapy caused sustained NK activity and prevented leukocyte drop	//	//	[145]
	Case series; add-on to chemotherapy, identifier not available, n = 3	Improvement of QoL	↑ NK cell activity	↓ immunosuppressive acidic protein	//	[146]
	Case series, add-on to post-operative hormone therapy, identifier not available, n = 20	Improvement of QoL	//	↑ IFNγ, ↑ IFNγ/L-10	//	[147]

**Table 3:** Score for the considered mushrooms related to their BC properties (*** more than 5 studies; ** 3–5 studies; * 1–2 studies; - no studies; modified from [29]).

Species	Type of Studies			Strength of Recommendation
	In Vitro	In Vivo	Clinical Studies	
***Agaricus bisporum***	***	**	*	**
***Antrodia cinnamomea***	***	***	*	**
***Cordyceps sinensis***	**	*	*	*
***Cordyceps militaris***	**	*	-	*
***Coriolus versicolor***	***	***	***	***
***Ganoderma lucidum***	***	***	***	***
***Grifola Frondosa***	***	**	***	***
***Lentinula edodes***	***	**	***	***
***Pleurotus ostreatus***	***	**	-	*

## Data Availability

All materials used in this review are available upon written request.
